# Excavatolide B Modulates the Electrophysiological Characteristics and Calcium Homeostasis of Atrial Myocytes

**DOI:** 10.3390/md15020025

**Published:** 2017-01-24

**Authors:** Hwong-Ru Hwang, Buh-Yuan Tai, Pao-Yun Cheng, Ping-Nan Chen, Ping-Jyun Sung, Zhi-Hong Wen, Chih-Hsueng Hsu

**Affiliations:** 1 Department of Marine Biotechnology and Resources, National Sun Yat-sen University, Kaohsiung 804, Taiwan; hwang.lin@msa.hinet.net (H.-R.H.); tai1337@hotmail.com (B.-Y.T.); 2 Division of Cardiology, Department of Medicine, E-Da Hospital, Kaohsiung 824, Taiwan; 3 Division of Cardiology, Department of Internal Medicine, Kaohsiung Veterans General Hospital, Kaohsiung 813, Taiwan; 4 Department of Traditional Medicine, Jianan Mental Hospital, Tainan 717, Taiwan; 5 Department of Physiology and Biophysics and Graduate Institute of Physiology, National Defense Medical Center, Taipei 114, Taiwan; pycheng@mail.ndmctsgh.edu.tw; 6 Department of Biomedical Engineering, National Defense Medical Center, Taipei 114, Taiwan; g931310@mail.ndmctsgh.edu.tw; 7 National Museum of Marine Biology and Aquarium, Pingtung 944, Taiwan; pjsung@nmmba.gov.tw; 8 Chinese Medicine Research and Development Center, China Medical University Hospital, Taichung 404, Taiwan; 9 Graduate Institute of Natural Products, Kaohsiung Medical University, Kaohsiung 807, Taiwan; 10 Doctoral Degree Program in Marine Biotechnology, National Sun Yat-Sen University and Academia Sinica, Kaohsiung 804, Taiwan; 11 Division of Cardiology, Tri-Service General Hospital, National Defense Medical Center, Taipei 114, Taiwan

**Keywords:** calcium regulation, cardiomyocyte, ionic currents, left atrium, sepsis

## Abstract

Severe bacterial infections caused by sepsis always result in profound physiological changes, including fever, hypotension, arrhythmia, necrosis of tissue, systemic multi-organ dysfunction, and finally death. The lipopolysaccharide (LPS) provokes an inflammatory response under sepsis, which may increase propensity to arrhythmogenesis. Excavatolide B (EXCB) possesses potent anti-inflammatory effects. However, it is not clear whether EXCB could modulate the electrophysiological characteristics and calcium homeostasis of atrial myocytes. This study investigated the effects of EXCB on the atrial myocytes exposed to lipopolysaccharide. A whole-cell patch clamp and indo-1 fluorimetric ratio technique was employed to record the action potential (AP), ionic currents, and intracellular calcium ([Ca^2+^]_i_) in single, isolated rabbit left atrial (LA) cardiomyocytes, with and without LPS (1 μg/mL) and LPS + EXCB administration (10 μM) for 6 ± 1 h, in order to investigate the role of EXCB on atrial electrophysiology. In the presence of LPS, EXCB-treated LA myocytes (*n* = 13) had a longer AP duration at 20% (29 ± 2 vs. 20 ± 2 ms, *p* < 0.05), 50% (52 ± 4 vs. 40 ± 3 ms, *p* < 0.05), and 90% (85 ± 5 vs. 68 ± 3 ms, *p* < 0.05), compared to the LPS-treated cells (*n* = 12). LPS-treated LA myocytes showed a higher late sodium current, Na^+^/Ca^2+^ exchanger current, transient outward current, and delayed rectifier potassium current, but a lower l-type Ca^2+^ current, than the control LA myocytes. Treatment with EXCB reversed the LPS-induced alterations of the ionic currents. LPS-treated, EXCB-treated, and control LA myocytes exhibited similar Na^+^ currents. In addition, the LPS-treated LA myocytes exhibited a lower [Ca^2+^]_i_ content and higher sarcoplasmic reticulum calcium content, than the controls. EXCB reversed the LPS-induced calcium alterations. In conclusion, EXCB modulates LPS-induced LA electrophysiological characteristics and calcium homeostasis, which may contribute to attenuating LPS-induced arrhythmogenesis.

## 1. Introduction

Severe sepsis is a leading cause of death worldwide, with myocardial dysfunction as one of the major predictors of morbidity and mortality [1]. However, the pathophysiology associated with sepsis is not fully elucidated, and the current treatment of sepsis is unsatisfactory. Hence, the development of clinically applicable, protective or therapeutic agents for sepsis, is critical. Lipopolysaccharide (LPS) is a potent inflammatory mediator that has been implicated in the pathogenesis of sepsis [2,3]. Treatment with LPS significantly attenuates left atrial (LA) dysfunction [4], and is a well-established animal model of systemic sepsis. Inflammation appears to be closely related to sepsis, whereas arrhythmia seems to create and sustain an inflammatory environment under sepsis. During LPS-induced sepsis, in many cells, including cardiomyocytes, vascular smooth muscle cells, endothelial cells, and macrophages, the over-production of nitric oxide (NO) can be induced by inducible NO synthase (iNOS) [5]. Elevated NO signaling has been shown to modulate many cardiac ionic channels, both deleterious and protective [6].

Excavatolide B (EXCB), a briarane-type diterpene compound, was isolated from the culture-type Formosan gorgonian *Briareum excavatum*, by the National Museum of Marine Biology & Aquarium in Taiwan [7]. EXCB possesses many anti-inflammatory, cytotoxic, and anti-tumor properties [8]. The anti-inflammatory activity of EXCB derives from the inhibition of mRNA expression of proinflammatory mediators, such as iNOS, c-Fos, and cyclooxygenase-2 (COX-2) [9]. Several epidemiological studies have confirmed that inflammation may increase the occurrence of arrhythmia [10]. Administrating LPS affects the calcium homeostasis and electrophysiological characteristics of atrial cardiomyocytes [11]. The role of inflammation in electrophysiological changes in atrial cardiomyocytes, and the effects of anti-inflammatory agents on sepsis, have been evaluated in various studies [12]. Arrhythmogenesis under sepsis could be reduced or inhibited by administering anti-inflammatory agents, in order to inhibit inflammation [13]. Growing evidence for the link between sepsis and inflammation indicates that pharmacological intervention with anti-inflammatory agents, to modulate inflammatory pathways, may be efficacious in the prevention of arrhythmogenesis in clinical practice [14].

However, it remains unclear whether EXCB directly regulates the calcium homeostasis and electrophysiological characteristics of LA cardiomyocytes under the influence of LPS, or functions indirectly through other anti-inflammatory mechanisms. Therefore, the present study examined whether EXCB can modulate LA electrical activities through calcium homeostasis, or regulate ionic currents in LPS-treated LA cardiomyocytes.

## 2. Results

### 2.1. Effects of EXCB on the Viability of LA Myocytes

**Figure 1:**
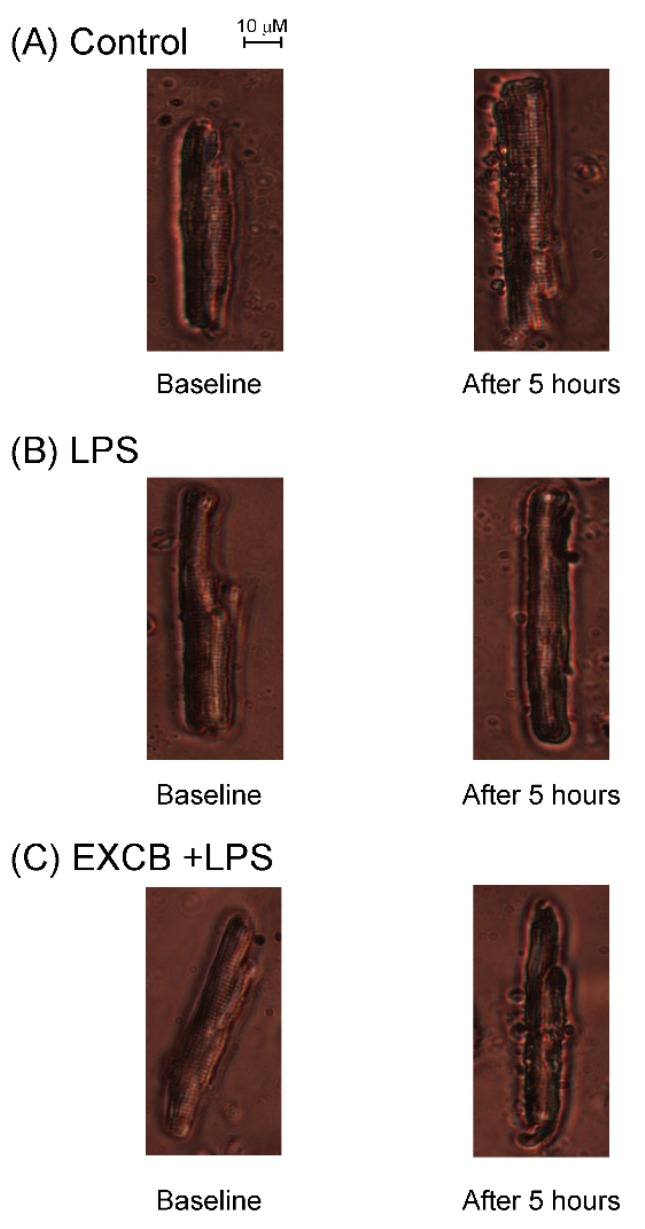
Effect of EXCB on the cell viability of control, LPS-treated, and LPS + EXCB-treated LA myocytes. LA myocytes from control and LPS-challenged rabbit with and without EXCB added after 5 h shown in panel (**A**) (control); (**B**) (LPS) and (**C**) (LPS + EXCB).

The results indicate that these drugs (EXCB 10 μM and LPS 1 μg/mL) did not affect the viability (Control group: 80% ± 7%; LPS group: 82% ± 5%; LPS + EXCB group: 64% ± 14%) of the LA myocytes, under such experimental circumstances (Figure 1).

### 2.2. Effects of EXCB on the Action Potential Morphology of LA Myocytes

To evaluate the effect of EXCB on the action potential morphology of LA myocytes, we used LPS (1 μg/mL) to pretreat LA myocytes, and added EXCB (10 μM) to measure the difference in the action potential duration (APD) at 20% (APD_20_), 50% (APD_50_), and 90% (APD_90_).

**Figure 2:**
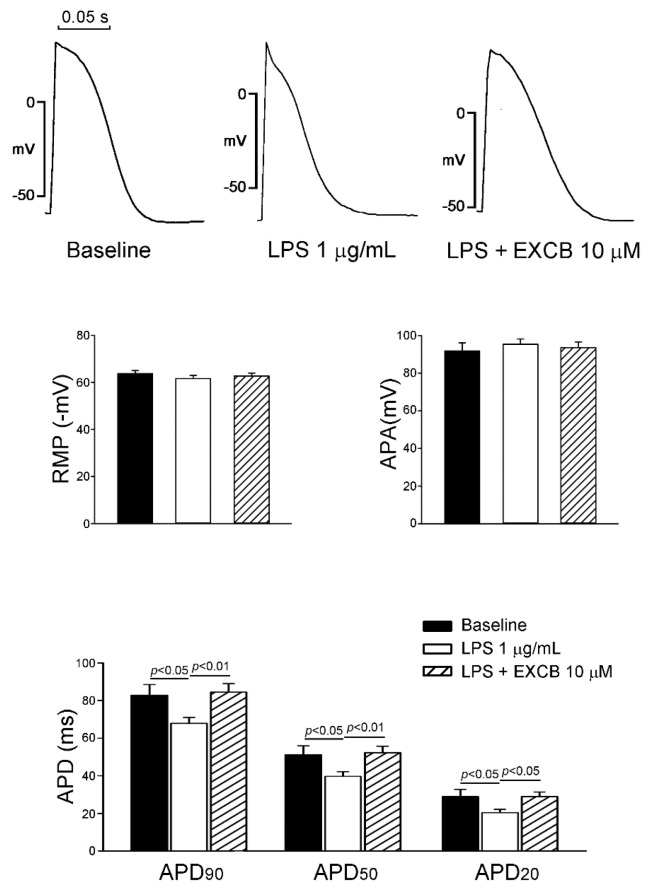
Effect of EXCB on the left atrial (LA) action potential morphology of control, LPS-treated and LPS + EXCB-treated LA myocytes. Examples and average data of the action potential from control (*n =* 11), LPS-treated (*n =* 12), and LPS + EXCB-treated (*n =* 14) LA myocytes. RMP resting membrane potential, APA action potential amplitude, APD_90_ 90% of action potential duration, APD_50_ 50% of action potential duration, APD_20_ 20% of action potential duration.

Figure 2 shows the AP morphology of the control, LPS-treated, and LPS + EXCB-treated LA myocytes. There was a significant difference in APD, but similarities in AP amplitude (APA), and resting membrane potential among the control, LPS, and LPS + EXCB groups. The LPS-treated LA myocytes had shorter APD_20_, APD_50_, and APD_90_, when compared with the controls. Moreover, APD_20_, APD_50_, and APD_90_ in the LPS + EXCB-treated LA myocytes, were longer than those in the LPS-treated LA myocytes.

### 2.3. Effects of EXCB on the Membrane Currents of LA Myocytes

This section details the effect of EXCB on the Na^+^ current (*I*_Na_), late sodium current (*I*_Na-Late_), l-type Ca^2+^ channel (*I*_Ca-l_), Na^+^/Ca^2+^ exchanger (NCX) current, transient outward current (*I*_to_), and delayed rectifier potassium current (*I*_K_).

**Figure 3:**
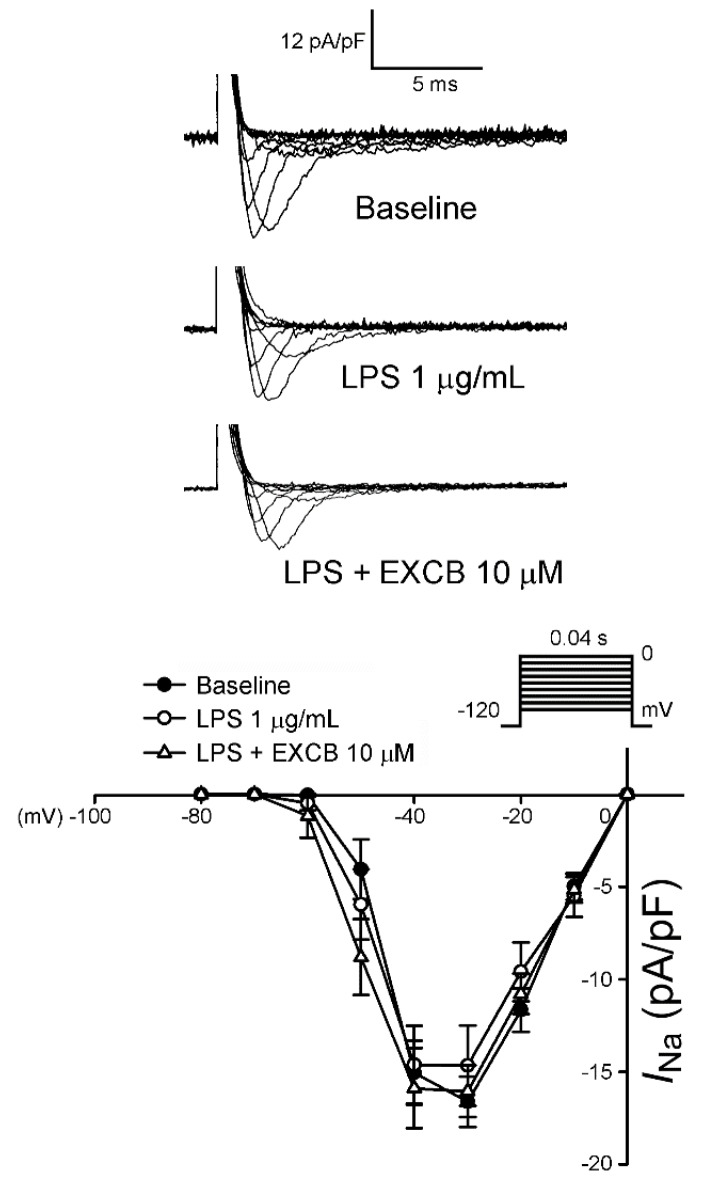
Effects of EXCB on Na^+^ current (*I*_Na_) in left atrial (LA) myocytes. Examples and the I-V relationship of the *I*_Na_ from control (*n* = 13), LPS-treated (*n =* 12), and LPS+EXCB-treated (*n =* 14) LA myocytes. The insets in the current traces show the various clamp protocols.

**Figure 4:**
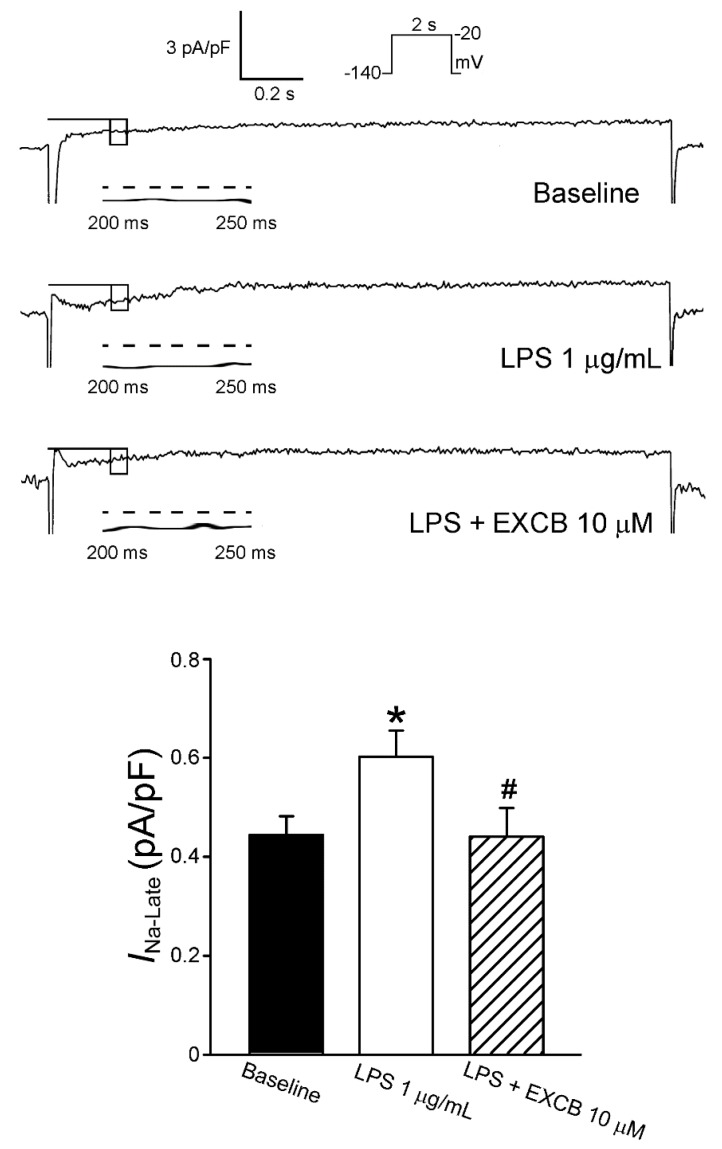
Effects of EXCB on late sodium current (*I*_Na-Late_) in left atrial (LA) myocytes. Examples and the average data of the *I*_Na-Late_ in control (*n =* 11), LPS-treated (*n =* 12), and LPS + EXCB-treated (*n =* 12) LA myocytes. The insets in the current traces show the various clamp protocols. * *p* < 0.05 versus control LA myocytes; ^#^ *p* < 0.05 versus LPS-treated LA myocytes. Data are presented as the mean ± S.E.M.

No significant difference was observed between the baseline values in *I*_Na_ among the control, LPS, and LPS + EXCB groups (Figure 3). Compared with the controls, the LPS-treated LA myocytes exhibited a significantly larger *I*_Na-Late_ (*p* < 0.05) (Figure 4). In addition, the *I*_Na-Late_ in the LPS + EXCB-treated LA myocytes was significantly smaller than that in the LPS-treated LA myocytes.

**Figure 5:**
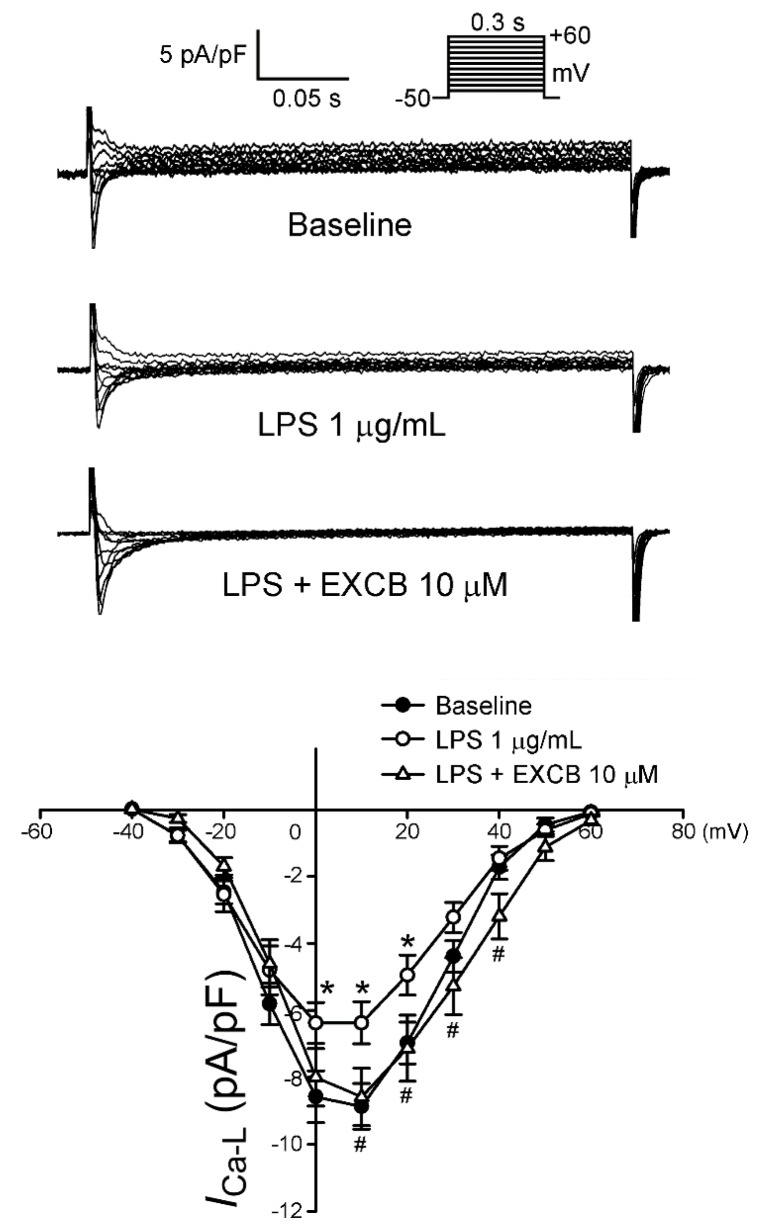
Effects of EXCB on the l-type Ca^2+^ channel (*I*_Ca-l_) in left atrial (LA) myocytes. The current traces and I-V relationship of *I*_Ca-l_ from control (*n =* 14), LPS-treated (*n =* 13), and LPS + EXCB-treated (*n =* 11) LA myocytes. The insets in the current traces show the various clamp protocols. * *p* < 0.05 versus control LA myocytes; ^#^ *p* < 0.05 versus LPS-treated LA myocytes.

As shown in Figure 5, *I*_Ca-l_ in the LPS-treated LA myocytes was significantly smaller than that in the controls, with a 28.1% decrease in the peak current (elicited from −50 to 10 mV). Compared with the LPS-treated LA myocytes, the LPS + EXCB-treated LA myocytes had a larger *I*_Ca-l_, demonstrating a 34.7% increase in the peak current (elicited from −50 to 10 mV).

**Figure 6:**
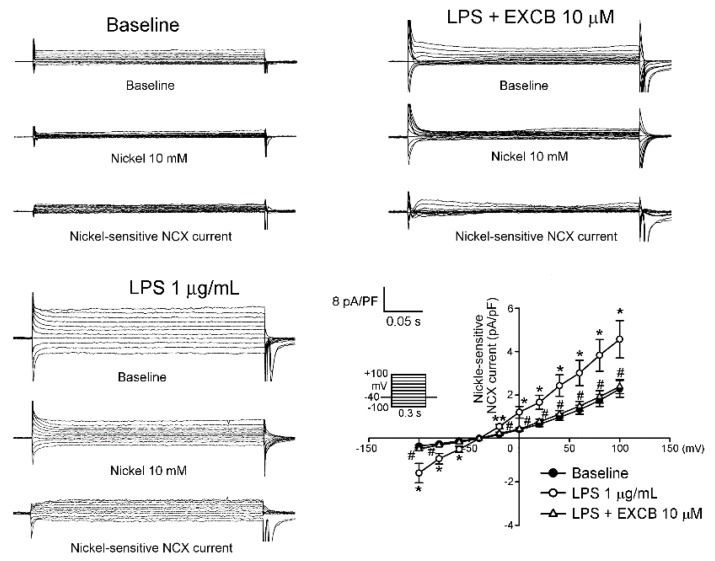
Effects of EXCB on the nickel-sensitive Na^+^/Ca^2+^ exchanger (NCX) in left atrial (LA) myocytes. The current tracings, I-V relationship of the nickel-sensitive NCX currents in the control (*n =* 9), LPS-treated (*n =* 11), and LPS + EXCB-treated (*n =* 10) LA myocytes. The insets in the current traces show the various clamp protocols. * *p* < 0.05 versus control LA myocytes; ^#^ *p* < 0.05 versus LPS-treated LA myocytes.

The forward and reverse modes of the NCX currents were larger in the LPS-treated LA myocytes (Figure 6) than in the controls, with a 50.2% and 76.7% increase in the peak current in the forward (elicited from −40 to 100 mV) and reverse (elicited from −40 to −100 mV) modes, respectively. However, the presence of EXCB reduced the forward and reverse modes of the NCX currents, with a 47.9% and 68.5% decrease in the peak current in the forward (elicited from −40 to 100 mV) and reverse (elicited from −40 to −100 mV) modes, respectively.

**Figure 7:**
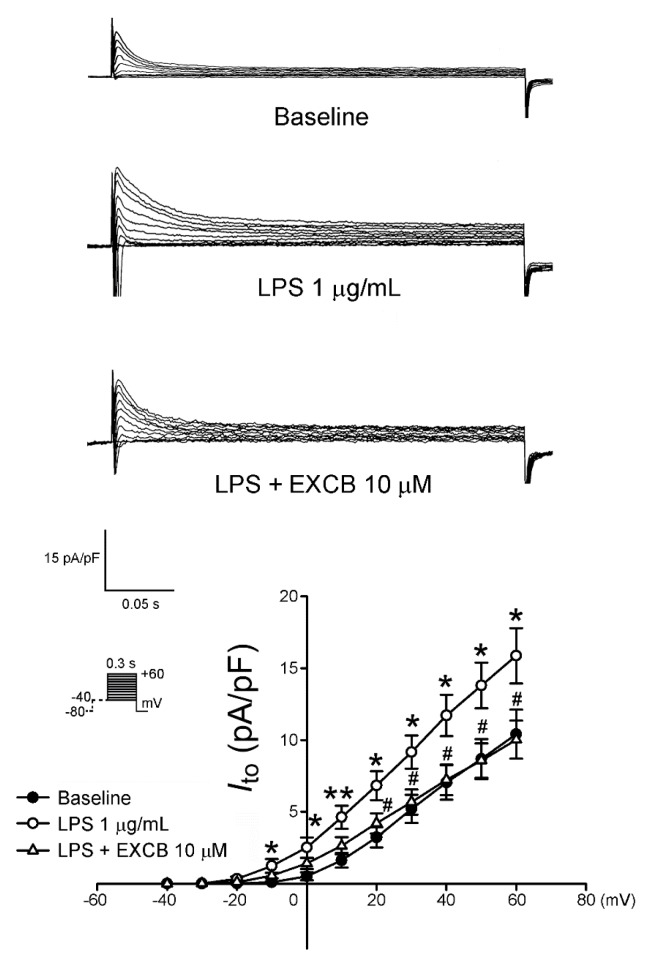
Effects of EXCB on the transient outward current (*I*_to_) in left atrial (LA) myocytes. Examples and I-V relationship of the *I*_to_ from control (*n =* 9), LPS-treated (*n =* 11), and LPS + EXCB-treated (*n =* 11) LA myocytes. The inset in the current traces shows the clamp protocol. * *p* < 0.05; ** *p* < 0.01 versus control LA myocytes; ^#^ *p* < 0.05 versus LPS-treated LA myocytes.

**Figure 8:**
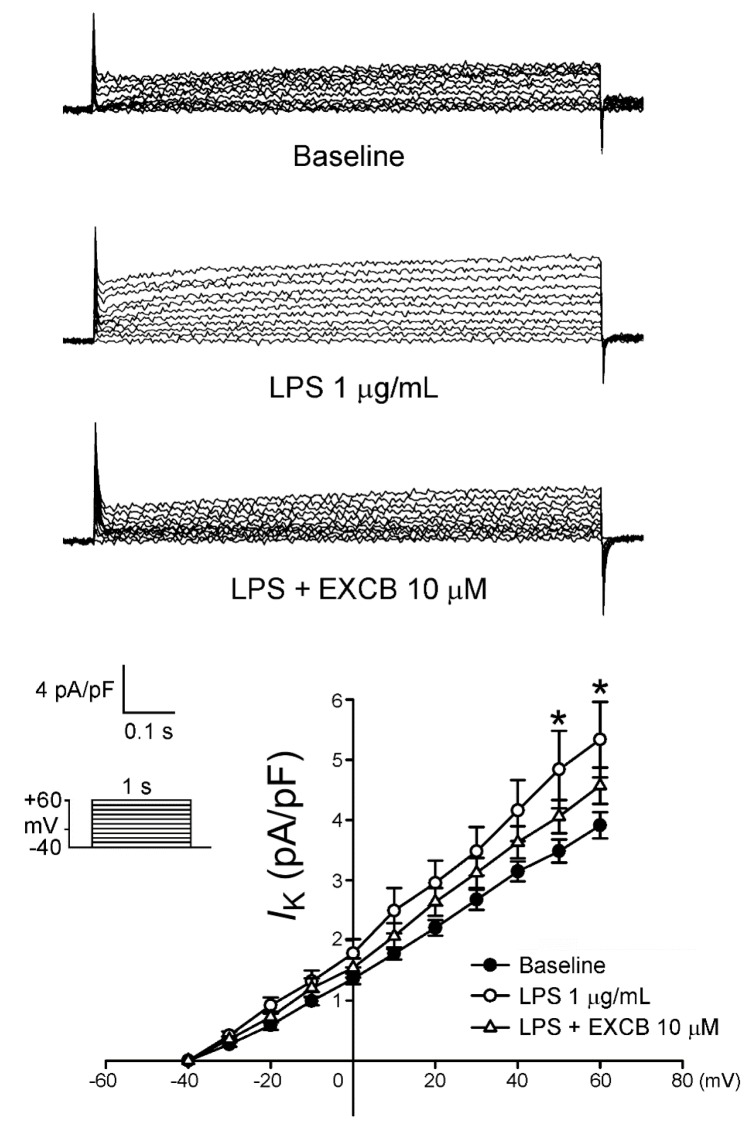
Effects of EXCB on the delayed rectifier potassium current (*I*_K_) in left atrial (LA) myocytes. Examples and I-V relationship of the *I*_K_ in the control (*n =* 11), LPS-treated (*n =* 9), and LPS + EXCB-treated (*n =* 9) LA myocytes. The inset in the current traces shows the clamp protocol. * *p* < 0.05 versus control LA myocytes.

Compared with the controls, the *I*_to_ of the LPS-treated LA myocytes was larger, exhibiting a 52.4% increase in the peak current (elicited from −40 to 60 mV). Moreover, the presence of EXCB reduced the *I*_to_, displaying a 36.8% decrease in the peak current (elicited from −40 to 60 mV; Figure 7). The LPS-treated LA myocytes had a larger *I*_K_ than the controls, with a 26.7% increase in the peak current (elicited from −40 to 60 mV), but the LPS-treated and LPS + EXCB-treated LA myocytes exhibited similar *I*_K_ values (Figure 8).

### 2.4. Effects of EXCB on Calcium Handling of LA Myocytes

**Figure 9:**
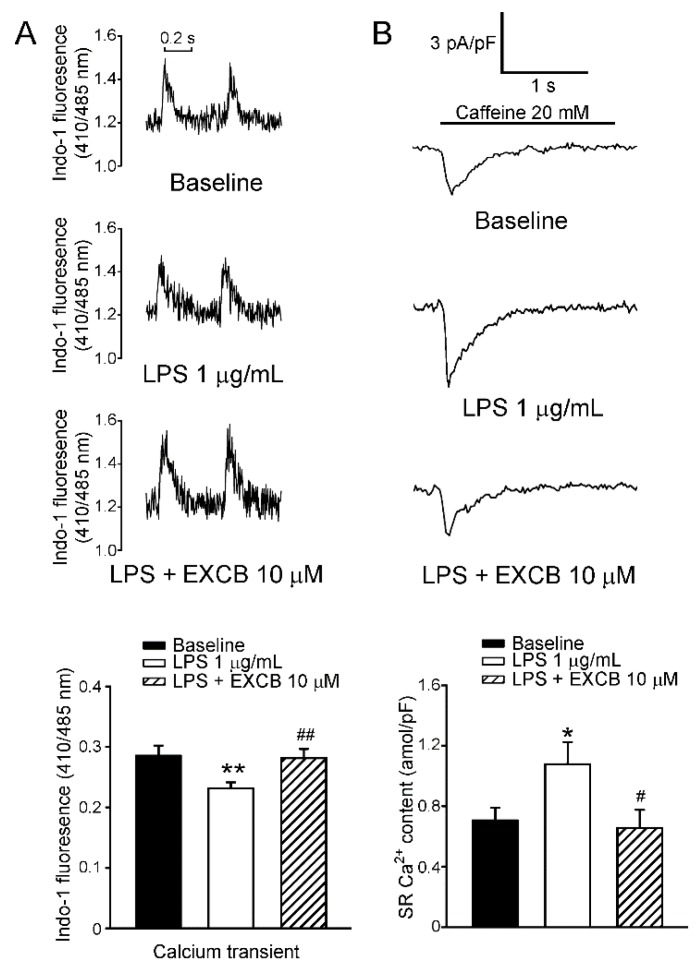
Effects of EXCB on the intracellular calcium and sarcoplasmic reticulum calcium content of the LA myocytes. (**A**) The tracings and average data from the [Ca^2+^]_i_ transient in the control, LPS-treated, and LPS + EXCB-treated LA myocytes; (**B**) The tracings of the caffeine-induced NCX currents and average data of SR calcium content from integrating the NCX current in the control (*n =* 11), LPS-treated (*n =* 10), and LPS + EXCB-treated (*n =* 10) LA myocytes. * *p* < 0.05, ** *p* < 0.01 versus the control group; ^#^ *p* < 0.05, ^##^ *p* < 0.01 versus the LPS group.

As shown in Figure 9A, the LPS-treated LA myocytes (*n =* 28) exhibited a smaller amplitude of intracellular calcium ([Ca^2+^]_i_) transients than the controls (*n =* 22). Following incubation with LPS ± EXCB (*n =* 27), the amplitude of the [Ca^2+^]_i_ transients further increased (*p* < 0.01 vs the LPS group). However, the sarcoplasmic reticulum (SR) Ca^2+^ content from integrating a caffeine-induced NCX current into the LPS-treated LA myocytes, was higher than that in the controls (Figure 9B). In addition, the SR calcium content, derived from integrating the caffeine-induced NCX current into the LPS + EXCB-treated LA myocytes, was significantly less than that in the LPS-treated LA myocytes.

## 3. Discussion

Based on our research, this study is the first to demonstrate that EXCB can modulate the electrophysiological characteristics and Ca^2+^ homeostasis of LPS-treated LA myocytes. In the present study, EXCB alleviates the effects of LPS on the LA action potential morphology, ionic currents, [Ca^2+^]_i_ transients, and SR Ca^2+^ content. The effects of EXCB on LPS-treated LA indicated that it might play a role in LA arrhythmogenesis during early stage tachyarrhythmia, and that inhibiting the calcium overload can reduce the persistence and genesis of AF.

AF is the most common arrhythmia encountered in clinical practice. Risk factors of AF include heart valve disease, heart failure, hypertension, and aging. Moreover, septic shock has been shown to be an independent risk factor for AF in postsurgical intensive care units [15,16]. At the cellular level, the arrhythmogenic substrate is characterized by a shortened APD without a plateau phase and poor adaptation to changes in heart rate [17]. Thus, LPS, a cell membrane surface component of Gram-negative bacteria, is vital for sepsis development. In the atrial myocytes of LPS-infused animal models, APD was shown to be significantly shortened [18]. Similarly, in the present study, the APD_20_, APD_50_, and APD_90_ of LPS-treated LA myocytes were all significantly shorter when compared with those of the controls. Meanwhile, this arrhythmogenics pattern, induced by LPS, was significantly reversed when the LPS-treated LA myocytes were co-treated with EXCB. These results suggest that EXCB may exert some anti-arrhythmogenic effects.

One of the major mechanisms of APD-shortening during AF is the reduction of *I*_Ca-l_. A reduction of nearly 70% in *I*_Ca-l_ has been consistently observed during AF, in both human atrial myocytes and experimental models [19,20,21]. Similarly, in the present study, the *I*_Ca-l_ was significantly reduced in the LPS-treated LA myocytes, when compared with the controls, and the effect of LPS on *I*_Ca-l_ was significantly reversed when the LPS-treated LA myocytes were co-treated with EXCB. The results of the present study reveal that the Ca^2+^ influx, specifically the Ca^2+^ current passed through the l-type Ca^2+^ channels of the sarcolemma reticulum, may be regulated using EXCB, further confirming the effect on the AP under the influence of LPS.

The *I*_Na-Late_ is the residual *I*_Na_, flowing after the peak *I*_Na_ during an AP event. Although it is a small sodium current under typical conditions, relative to the peak *I*_Na_, it is sufficiently able to affect the APD during the AP plateau, and the sodium ion flow over hundreds of milliseconds during an AP event, contributes more towards consequent sodium loading, than to the large transient peak *I*_Na_ [22]. Previous studies have shown that *I*_Na-Late_ plays a crucial role in the arrhythmogenic APs of the ventricles and atria [23,24,25,26]. We found that the *I*_Na_ flux did not significantly differ among the control, LPS-treated, and LPS + EXCB-treated LA myocytes, and that the *I*_Na-Late_ current density in the LPS + EXCB-treated LA myocytes was significantly reduced, relative to that in the LPS-treated LA myocytes.

An increase in *I*_Na-Late_ in the heart can lead to arrhythmia by affecting NCX, causing a calcium overload [27,28], and the NCX plays a critical role in atrial arrhythmogenesis [29]. The NCX performs electrogenic ionic exchange of sodium and calcium ions across the plasma membrane, in either the Ca^2+^ efflux forward or Ca^2+^ influx reverse modes, depending on the driving force of the electrochemical gradients of the substrate ions. An NCX-mediated increase in calcium entry, or decrease in calcium exit, due to a rise in intracellular sodium ([Na^+^]_i_), results in calcium overloading of the SR, leading to the mechanical and electrical dysfunction of myocytes [30]. The suppressed *I*_Na-Late_ by EXCB decreases the [Na^+^]_i_, which changes the Na^+^/Ca^2+^ flux direction. Moreover, previous study has shown that late Na^+^ channel gating is regulated by Ca^2+^/calmodulin (CaM)/Ca^2+^-dependent CaM-kinase II (CaMKII) [31]. Accordingly, EXCB prevents intracellular Ca^2+^ overload, which may reduce arrhythmogenesis [32,33,34]. The decrease in the *I*_Na-Late_ and NCX currents by the EXCB + LPS-treated LA myocytes, may be related to the inhibition of CaMKII. Thus, the reduction of *I*_Na-Late_, but not *I*_Na_, in the LPS + EXCB-treated LA myocytes, suggests a more selective sodium channel blockade by EXCB in LA arrhythmogenesis. In the present study, both of the *I*_Na-Late_ and NCX currents were significantly increased in the LPS-treated LA myocytes, and these patterns were significantly attenuated by co-treatment with EXCB.

In addition, the [Ca^2+^]_i_ content was significantly reduced and the SR Ca^2+^ content was increased in the LPS-treated LA myocytes, and these effects were significantly reversed by co-treatment with EXCB. The reduction in the SR Ca^2+^ content in the LPS + EXCB-treated LA myocytes, may be due to the suppression of NCX currents with EXCB, in both the forward and reverse modes. A decrease in NCX currents and *I*_Na-Late_, can reduce [Ca^2+^]_i_ on LA, which may attenuate Ca^2+^ overload-induced activity [29]. Therefore, the effects of EXCB on calcium handling may have contributed to the decreased SR calcium content in the LPS-treated LA myocytes, and may have modified the LA electrophysiological property.

In addition to the change in *I*_Ca-l_, *I*_K_ is a major component that determines the timing domain of the repolarization of myocytes; an increase in *I*_K_ was shown to contribute to the shortening of the APD in atrial myocytes, obtained from septic guinea pigs [18]. *I*_to_ and *I*_K_ have also been shown to play crucial roles in the repolarization of the atrium in humans [35,36]; thus, both *I*_to_ and *I*_K_ have been targets in anti-AF studies [37,38]. Similarly, in the present study, both *I*_to_ and *I*_K_ were significantly increased in the LPS-treated LA myocytes, but significantly reduced when the LPS-treated LA myocytes were co-treated with EXCB. These findings may further confirm the consistency of the effect of LPS on the APD. Moreover, the findings reveal that the anti-arrhythmogenic effect of EXCB on LPS-treated LA cardiomyocytes, may be associated with the suppression of transient outward currents and delayed rectifier potassium currents. Because *I*_to_ and *I*_K_ are major determinants of early repolarization, EXCB-impaired *I*_to_ and *I*_K_ slowed early repolarization, which may account for the reduction in spontaneous LA electrical activity.

In cases of sepsis, pro-inflammatory stimuli lead to overexpression of iNOS in many vital organs. Excess NO generated by iNOS is implicated in the pathophysiology of sepsis progression [39]. Recent studies have suggested that reactive nitrogen species, including NO, peroxynitrite, and nitrogen dioxide, can modulate the structure and function of the ion channel through the mediating protein tyrosine [40]. Thus, we assume that the iNOS-induced, over-production of NO in LPS-treated LA myocytes may act as a means of increasing tyrosine nitration of l-type Ca^2+^ channels, leading to impaired channel function. Speculation is supported by another report [41], and our results indicate that the APD and *I*_Ca-l_ were significantly reduced in LPS-treated LA myocytes. Moreover, EXCB has been shown to exhibit some anti-inflammatory effects through inhibition pro-inflammatory mediators, such as iNOS and COX-2. LPS-induced changes of electrophysiological characteristics and calcium homeostasis can be alleviated by EXCB. Although the incubation period of LPS is not long enough to induce arrhythmogenic-triggered activity, the results in the present study imply that EXCB may possibly reduce the risk of LPS-induced LA arrhythmogenesis.

## 4. Experimental Section

### 4.1. Materials

EXCB was isolated from the culture type of soft coral Formosan Gorgonian *Briareum excacatum*, by the National Museum of Marine Biology & Aquarium (NMMB, Pingtung, Taiwan).

### 4.2. Isolation of Single LA Myocytes

All experiments conformed to the institutional Guide for the Care and Use of Laboratory Animals, and were approved by a local ethics review board (IACUC-13-267) and the *Guide for the Care and Use of Laboratory Animals* [42]. Male rabbits, weighing 1 to 2 kg, were anesthetized intravenously and injected with sodium pentobarbital (100 mg/kg). The hearts were immediately removed and mounted on a Langendorff apparatus after anesthetization, in order to perform perfusion in 95% O_2_ oxygenated normal Tyrode’s solution, at 37 °C, containing (mM): NaCl, 137; glucose, 11; HEPES, 10; KCl, 5.4; CaCl_2_, 1.8; MgCl_2_, 0.5, and the pH was adjusted to 7.4 with NaOH. After the hearts were cleaned of all traces of blood, this perfusate was replaced with oxygenated Ca^2+^-free Tyrode’s solution, containing collagenase type I (300 units/mL) (Sigma Chemical, St. Louis, MO, USA) and protease type XIV (0.25 units/mL) (Sigma Chemical, St. Louis, MO, USA), for about 8–12 min, in order to perform enzymatic dispersion [23]. The LA was then excised into small pieces and gently shaken in 50 mL of Ca^2+^-free oxygenated Tyrode’s solution, until single cardiomyocytes were isolated. The solution was then gradually changed to normal oxygenated Tyrode’s solution. In this study, EXCB was obtained from the National Museum of Marine Biology & Aquarium (NMMBA), Kaohsiung, Taiwan. The rabbit LA myocytes were allowed to stabilize in the bath for at least 30 min, before performing experiments.

### 4.3. Electrophysiological Study

All of the whole-cell patch-clamp recordings were made from the single LA myocytes in the control and in the presence of lipopolysaccharides (LPS, 1 μg/mL) [43], with and without EXCB (10 μM) [7], by using an Axopatch 1D amplifier (Axon Instruments, Sunnyvale, CA, USA) at 35 ± 1 °C. During this study, almost all of the experiments were unpaired, carried out on separate groups of cells: under control conditions and after incubation of cells for 6 ± 1 h in the presence of LPS, with and without EXCB. The thin-walled borosilicate capillary tubes (o.d. 1.8 mm) were used to fabricate microelectrodes with tip resistances of approximately 3–5 MΩ. Before the formation of a stable membrane-pipette seal, the tip potentials were zeroed in Tyrode’s solution. Junction potentials between the bath and pipette solution (9 mV) were also corrected for AP recordings. Action potentials were recorded in current-clamp mode, and ionic currents were measured in the voltage-clamp mode. A small hyperpolarizing step from a holding potential of −50 mV, to a testing potential of −55 mV for 80 ms, was conducted at the beginning of each experiment. The total cell capacitance was determined by the the area obtained under the capacitive currents, which was divided by the applied voltage step. Normally, 60%–80% series resistance (Rs) was electronically compensated. The RMP was obtained during the period between the last repolarization and the onset of the subsequent AP. The APA was obtained from the measurement of RMP, to the peak of the AP depolarization. All AP durations were measured at 20% (APD_20_), 50% (APD_50_), and 90% (APD_90_) repolarization of the amplitude stimulated with a rate of 2 Hz in all preparations.

The filling solution used in the micropipettes consisted of (mM) CsCl, 130; HEPES, 10; MgATP, 5; Na_2_ phosphocreatine, 5; MgCl_2_, 1; NaGTP 0.1 (adjusted to pH 7.2 with CsOH) for the experiments on the *I*_Ca-l_; with a solution consisting of (mM) CsCl, 133; TEACl, 20; EGTA, 10; HEPES, 5; MgATP, 5; NaCl, 5 (adjusted to pH 7.3 with CsOH) for the *I*_Na_. These contained (mM) NaCl, 10; CsCl, 130; EGTA, 5; HEPES, 5; glucose, 5; ATP-Mg, 5 for the *I*_Na-Late_; containing (in mM) CsCl, 110; NaCl, 20; MgCl_2_, 0.4; CaCl_2_, 1.75; TEACl, 20; HEPES, 10; BAPTA, 5; glucose, 5; MgATP, 5 (adjusted to pH 7.25 with CsOH) for the experiments on the NCX current. Those contained (mM) K aspartate, 110; KCl, 20; HEPES, 10; MgATP, 5; Na_2_ phosphocreatine, 5; MgCl_2_, 1; EGTA, 0.5; LiGTP 0.1, and pH was adjusted to 7.2 with KOH for the experiments on the AP and potassium currents.

The *I*_Na_ was recorded during depolarization, from a holding potential of −120 mV, to testing potentials ranging from −90 to +60 mV in 10 mV steps for 40 ms, at a frequency of 3 Hz and a room temperature of 25 ± 1 °C unless otherwise specified. External solution was used, containing (mM): CsCl, 133; NaCl, 5; HEPES, 5; glucose, 5; MgCl_2_, 2; CaCl_2_, 1.8; nifedipine 0.002, with pH of 7.3.

The *I*_Na__-Late_ was recorded at room temperature (25 ± 1 °C) with external solution containing (mM): NaCl, 140; CsCl, 5; glucose, 5; HEPES, 5; MgCl_2_, 2; CaCl_2_, 1.8; nicardipine, 0.002. The amplitude of the *I*_Na__-Late_, at a voltage of −20 mV, was measured as the mean current amplitude between 200 ms and 250 ms, after the membrane was depolarized by a 2000-ms period from −140 to −20 mV.

The *I*_Ca-l_ was measured as an inward current during depolarization from a holding potential of −50 mV, to testing pulses ranging from −40 to +60 mV in 10 mV steps for 300 ms, at a frequency of 0.1 Hz by means of a perforated patch clamp with amphotericin B. The NaCl and KCl in the normal Tyrode’s solution were replaced by TEACl and CsCl, respectively. In order to avoid the ‘run-down’ effects, *I*_Ca-l_ was measured on the peak inward current, and the current at the end of each test pulse.

The NCX current was elicited by test potentials between −100 to +100 mV, from a holding potential of −40 mV for 300 ms, at a frequency of 0.1 Hz. The amplitudes of the NCX current were measured as 10 mM nickel-sensitive currents. The external solution (mM) contained NaCl, 140; glucose, 10; HEPES, 5; CaCl_2_, 2; MgCl_2_, 1, strophanthidin (10 μM), nitrendipine (10 μM) and niflumic acid (100 μM), and the pH was adjusted to 7.4.

The *I*_to_ was performed with a double-pulse protocol. A 30-ms pre-pulse ranging from −80 to −40 mV was used to inactivate the sodium channels (*I*_Na_), followed by a 300 ms test pulse to +60 mV in 10 mV steps at a frequency of 0.1 Hz. CdCl_2_ (200 μM), which was added to the bath solution to inhibit *I*_Ca-l_. The *I*_to_ was measured as the difference between the peak outward current and the steady-state current.

The *I*_K_ was obtained from the outward current at the end of 1 s and a depolarization from −40 to +60 mV in 10 mV steps, at a frequency of 0.1 Hz during the infusion of CdCl_2_ (200 μM) and 4-aminopyridine (2 mM), was initiated in the bath solution to eliminate the involvement of other ionic currents.

### 4.4. Measurement of the Changes in the Intracellular Calcium Concentration

The [Ca^2+^]_i_ was recorded using a fluorimetric ratio technique (indo-1 fluorescence) in an isolated, single LPS-treated, LPS + EXCB-treated, and control LA myocyte, as described previously. The fluorescent indicator indo-1 was loaded by incubating the myocytes at room temperature for 30 min, with 10 μM of indo-1/AM (Sigma Chemical, St Louis, MO, USA). The LA myocytes were then perfused with Tyrode’s solution at 35 ± 1 °C for at least 20 min, in order to wash out the extracellular indicator and to allow for the intracellular de-esterification of the indo-1. The background and cell autofluorescence were canceled out by zeroing the output of the photomultiplier tubes using cells without indo-1 loading. A UV light of 360 nm with a monochromator was used for the excitation of the indo-1 by a xenon arc lamp, controlled by the microfluorimetry system (OSP100-CA, Olympus, Tokyo, Japan), and the excitation light beam was directed into an inverted microscope (IX-70, Olympus, Tokyo, Japan). The fluorescence signals emitted from the indo-1/AM-loaded myocytes were digitized at 200 Hz. The ratio of the fluorescence emission at 410 and 485 nm (R410/485) was used as the index of the [Ca^2+^]_i_. This approach avoided any uncertainties resulting from the calibration of the fluorescent calcium indicators. The [Ca^2+^]_i_ transients were measured during a 2 Hz field stimulation, with 10 ms square-wave pulses at double threshold strength, and were calculated from the difference of the peak systolic and diastolic [Ca^2+^]_i_ transients. The fluorescence ratio data were processed and stored on a computer using the relevant software (OSP-SFCA, Olympus, Tokyo, Japan). The sarcoplasmic reticulum (SR) Ca^2+^ content was measured by integrating the NCX current from rapidly adding caffeine (20 mM) into the cells during rest, with the membrane potential clamped to −40 mV. The time integral of the NCX current was converted to the molarity of the calcium ions released from the SR [23].

### 4.5. Statistical Analysis

All continuous variables are expressed as the mean ± S.E.M. The differences between the control, LPS, and LPS + EXCB-treated LA myocytes, were compared using the Mann-Whitney rank sum unpaired *t*-test, depending on the outcome of the normality test. A *p* value of less than 0.05 was considered statistically significant.

## 5. Conclusions

The effects of EXCB on LPS-induced LA arrhythmogenesis may be due to a decrease in *I*_Na-Late_, NCX current, *I*_to_, *I*_K_, and SR Ca^2+^ release. EXCB counteracts the effect of LPS-induced change in ion currents and APD. Therefore, our data demonstrate that EXCB may be a potential candidate for the management of septic atrial arrhythmogenesis.
